# Superior Cluneal Neuralgia Treated With Wireless Peripheral Nerve Stimulation

**DOI:** 10.7759/cureus.23711

**Published:** 2022-03-31

**Authors:** Gaurav Chauhan, Isaiah Levy, David DeChellis

**Affiliations:** 1 Anesthesiology and Perioperative Medicine, University of Pittsburgh Medical Center Presbyterian, Pittsburgh, USA; 2 Physical Medicine and Rehabilitation, University of Pittsburgh Medical Center, Pittsburgh, USA; 3 Pain Medicine, Heritage Valley Kennedy Pain Treatment Center, Pittsburgh, USA

**Keywords:** wireless implantable device, neuromodulation, lower back pain, peripheral nerve stimulator, cluneal neuralgia

## Abstract

Superior cluneal neuralgia (SCN) can often be misdiagnosed when evaluating a patient with low back pain (LBP). The pathomechanics of SCN can range from direct injury following surgeries or trauma to myofascial compression due to abnormal muscle tone or fibrosis. The authors present a case of SCN in a 65-year-old male that persisted for three years following a laminectomy complicated by retained hardware and subsequent fibrosis. The patient’s diagnosis was confirmed with a diagnostic nerve block with significant pain relief after initial misdiagnosis and unsuccessful interventions targeting other possible pain generators. He ultimately underwent a successful peripheral nerve stimulation (PNS) trial and implantation with significant long-term pain relief. This case report entails the need to consider SCN in the differential for low back pain and the successful utilization of PNS for treatment.

## Introduction

Low back pain (LBP) is a vexing clinical syndrome to live with as a patient and a challenge to diagnose as a physician. An initial broad differential diagnosis is required to avoid missing several possible pain generators. Superior cluneal neuralgia (SCN) can be an underdiagnosed etiology of low back pain. The superior cluneal nerve is a sensory nerve that provides sensory innervation to the posterior iliac crest and the skin overlying the superior portion of the gluteus maximus. The incidence of LBP resulting from SCN ranges between 1.6% and 14%, and 50% of patients with SCN might report associated leg symptoms [1,2]. Current literature reports that patients with SCN can present with symptoms ranging from limitations in lumbar motion to radiating pain into the legs and antalgic gait, leading to misdiagnosis and the risk of unnecessary spine surgeries [2]. This case report entails considering SCN in the differential for low back pain and the successful utilization of peripheral nerve stimulation (PNS) for treatment.

## Case presentation

The patient was a 65-year-old male with a body mass index of 32 kg/m^2^ and a past medical history of hypertension and well-controlled type 2 diabetes mellitus who presented to the chronic pain clinic due to three years of LBP. The patient consented for this case report to be published. The patient underwent lumbar laminectomy and fusion at L3 to L5 levels three years prior to initial presentation, which was complicated by a retained needle in the left supra-gluteal region. The patient underwent debridement surgery to retrieve the needle. The surgical incision extended transversely from the left superior gluteal region to the iliac crest, 5 cm anterior to the posterior superior iliac spine. Two weeks after his procedure, the nature of the pain changed from sharp pain to burning, stabbing pain over the surgical site that radiated to the lateral part of his left hip, thigh, and groin. The pain was constant and exacerbated upon sitting, walking, or rubbing clothes over the skin and was worse while wearing tight garments or belts around the waist. He denied any weakness in his lower extremities. The patient rated his pain as 6-8/10 on a numeric rating scale (NRS). He further reported that the pain adversely affects his quality of life and exacerbates his anxiety.

The patient underwent lumbar X-rays (flexion-extension and complete lumbar spine) that reported stable lumbar fusion and hardware anomalies. He received an ultrasound-guided trigger point injection with minimal relief of his symptoms. Three months after developing his symptoms, the patient started complaining of tingling, numbing, electric shock-like pain traveling to the front of the thigh. The patient reported that this new pain was intermittent and exacerbated on getting up from sitting, squatting down, changing position in the bed, or prolonged walking. There were no alleviating factors for this pain, and it coexisted with his chronic left hip pain. The subsequent lumbar magnetic resonance imaging (MRI) reported a new disc herniation on the left side at L2-L3 levels, sparing the exiting L2 spinal nerve. The patient underwent a transforaminal epidural steroid injection at L2 with 0.25% bupivacaine and 10 mg dexamethasone, which was successful in alleviating the leg pain. Consequently, the patient enrolled in physical therapy for core strengthening. The patient failed to continue the physical therapy sessions due to chronic left hip pain. The patient then underwent imaging of the left hip that reported moderate hip arthrosis. The patient received a left hip steroid injection and a greater trochanteric bursa injection without any relief from his chronic left hip pain. He again underwent trigger point injections, lumbar epidural steroid injections, greater trochanter bursa injections, sacroiliac joint injections, and intra-articular hip injections over three years at an outside pain clinic without any significant relief in his pain. The patient failed to achieve significant pain relief with 4% lidocaine patches, gabapentin (1200 mg three times daily), 50 mg nortriptyline at bedtime, and nonsteroidal anti-inflammatory agents.

After three years of unsuccessful treatments, paraspinal trigger point, lumbar disc pathology or subsequent radiculopathy, greater trochanteric pain syndrome, sacroiliac joint dysfunction, and intra-articular hip pathology were deemed less likely primary generators of his pain. The patient presented to the author’s pain clinic three and a half years after symptoms appeared. The patient described his pain as sharp, burning, and lancinating and rated 10/10 on the NRS scale. The patient reported that he has trouble sitting for prolonged periods, and pain interferes with his quality of life. He further informed that it was a significant stressor in his life post-retirement. The patient reported using 4% lidocaine patches over the painful sites, 200 mg Lyrica three times daily, and 75 mg nortriptyline at bedtime. After taking the medication, his pain decreased from a 10 to between 6 and 8 on the NRS scale. The physical examination revealed hyperalgesia over the superior gluteal region, extending to the iliac crest laterally. There were no motor or reflex changes observed. The patient had an antalgic gait due to pain and was wearing loose clothes. The patient was scheduled for a superior cluneal nerve block.

**Figure 1:**
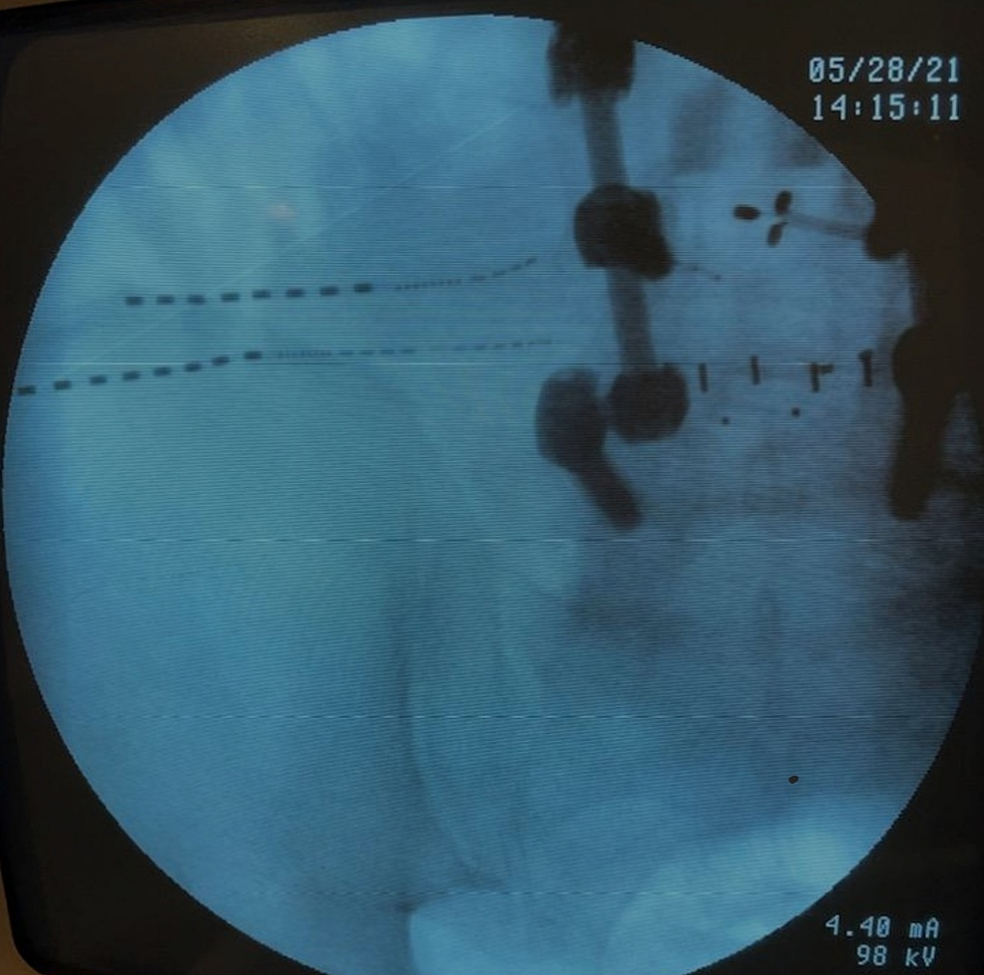
Stimwave trial lead placement over the left iliac crest.

The patient underwent a diagnostic superior cluneal nerve block with 0.25% bupivacaine and 40 mg Depo-Medrol, which provided him with 100% pain relief for two weeks. The pain came back to baseline four weeks after the nerve block. PNS was considered a long-term option due to the significant effect on the patient’s quality of life, the duration of his symptoms, and the substantial improvement with a diagnostic nerve block. Upon the initial trial utilizing a wirelessly powered PNS system for SCN, as shown in Figure 1, the patient reported 100% resolution of his symptoms.

The patient was then scheduled for a permanent implant with a Stimwave PNS system (StimwaveTechnologies, Pompano Beach, FL, USA). 

The patient was placed in the prone position. The anatomic landmarks of the patient were identified with fluoroscopy, marked with a marking pen. The skin was prepped widely, and a strict sterile technique was utilized throughout. The skin and subcutaneous tissue were infiltrated with 15 mL of 1% lidocaine. Two initial incisions were made superficially through the dermal layer lateral to the spinous processes at the L4-L5 levels on fluoroscopy. Hemostasis was maintained with monopolar cautery. Two 6-inch, 14-gauge introducers were threaded through the incision sites and placed at the location of the left superior clinical nerves, both just superior to the left iliac crest in addition to just posterior to the proximal aspect of the left iliac crest. At this point, the stylets of the introducers were removed, and two Stimwave peripheral nerve stimulator four contact leads were placed through the introducers. Subsequently, the introducers were removed under fluoroscopic guidance to assure continued lead placement without migration. At this point, the blank stylets of the PNS leads were removed, and neuroreceiver stylets were placed into each of the leads.

**Figure 2:**
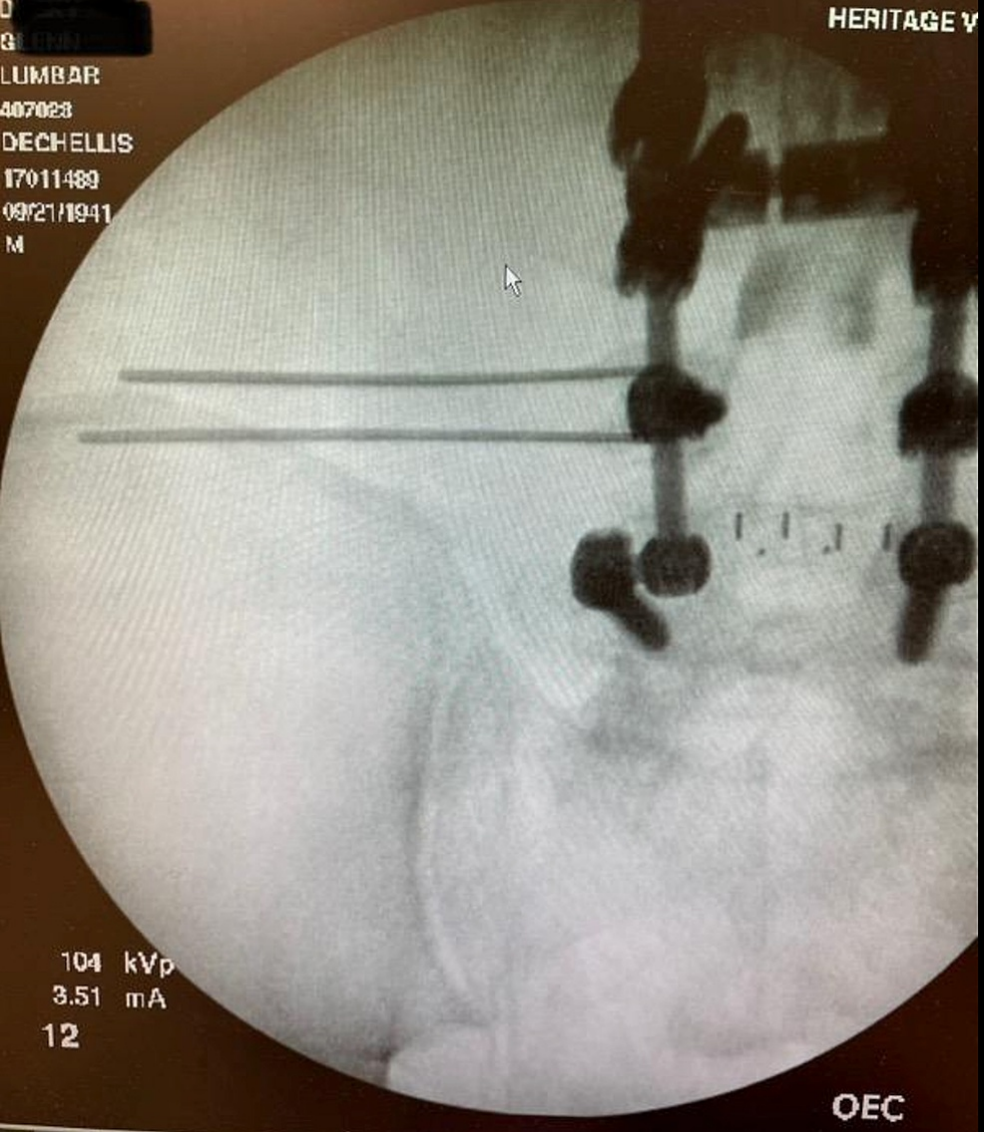
Thirteen-gauge introducer Tuohy needles placed over the left iliac crest.

Stimulation was then tested to capture the patient’s region of pain in the lumbosacral region. At this point, two neuroreceiver pockets were then made rostrally to the initial incision sites. At this point, the two leads were then tunneled from their initial entry site incisions to the neuroreceiver pockets using 13-gauge introducers (Figure 2).

**Figure 3:**
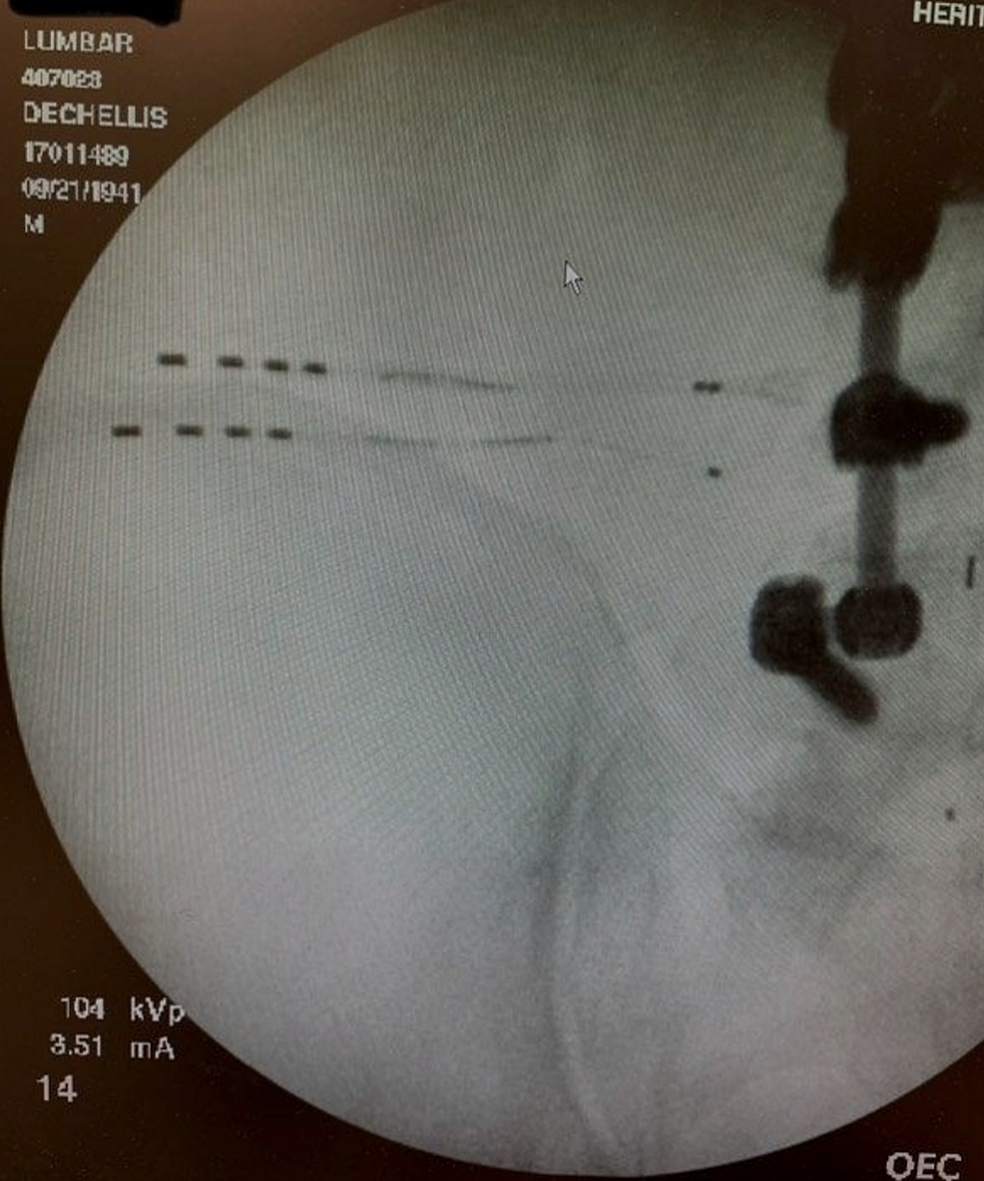
Two Stimwave wireless peripheral stimulator leads placed over the left iliac crest. The coils are made at the end of the leads and buried under the skin.

The two leads were then anchored to the deep fascia using a 1-0 Prolene suture. Neuroreceiver coils were then made at the end of the two leads, after which the neuroreceiver coils were buried into the neuroreceiver pockets. The subdermal layer was then closed using 2-0 Vicryl buried sutures in addition to superficially using 4-0 Monocryl running stitch (Figure 3).

There were no unwanted complications or paresthesia observed during the procedure. An abdominal binder was placed prior to transferring out of the operating room. The patient was then taken to the PACU in stable condition. The patient was discharged home from the outpatient surgery department in a stable condition.

At six months after the PNS implantation, the patient reported more than 80% improvement in pain and more than 80% improvement in function. He commented that now he can sit along with his friends without obvious discomfort.

## Discussion

The superior cluneal nerve usually derives from the dorsal rami of the upper lumbar spinal nerve roots (L1-L3) but with possible contributions ranging from T12 to L5 [3-5]. The superior cluneal nerve traverses through the psoas major and paraspinal muscles, posterior to the quadratus lumborum, and then through the inferior latissimus dorsi [3], eventually dividing into the medial, middle, and lateral branches to provide sensory innervation to the posterior iliac crest and the skin overlying the superior portions of the gluteus maximus. Anatomic dissection has revealed that, in 55% of cadavers, the medial superior cluneal nerve passes through an osteofibrous tunnel between the iliac crest and the thoracolumbar fascia when traversing over the iliac crest, which can be a possible cause of superior cluneal nerve entrapment [5].

The most common etiology of SCN is iatrogenic, such as autologous bone graft harvest from the iliac crest, spinal surgical procedures, debridement procedures, or muscle flap surgeries [1,3,6], as well as conditions that result in abnormal muscle tone leading to myofascial compression, ranging from pain-mediated maladaptive posturing due to lumbar spinal stenosis, disc herniations, scoliosis, and fractures to spasticity and rigidity in conditions such as Parkinson’s disease [1,7]. The patient, in this report, developed SCN due to the debridement procedure and resultant fibrosis, leading to the entrapment of the superior cluneal nerves.

Physical examination can help elucidate whether the superior cluneal nerve branches are primarily involved in a patient’s LBP. Examination maneuvers such as maximal tenderness and possible paresthesia or allodynia along the superior aspect of the gluteus maximus may increase the likelihood of superior cluneal nerve involvement. The reproduction of pain on stretching muscles along the superior cluneal nerve plane, with maneuvers such as lateral bending and stretching of the ipsilateral quadratus lumborum or full flexion of the ipsilateral hip and knee, can also increase the likelihood of SCN pathology [6,7].

The treatment options for SCN include local nerve blocks and neuroablative procedures such as phenol neurolysis or radiofrequency ablation [8-10]. In more refractory cases, surgical decompression can be utilized to decompress the distal superior cluneal nerve branches or the contributing lumbar radicular segments [10,11]. PNS has been explored more recently as an option for patients with refractory SCN. Abd-Elsayed demonstrated the successful utilization of a wireless PNS system in five patients with different types of neuralgias, including cluneal neuralgia. This report adds to the evidence that PNS can successfully be utilized for patients with LBP due to SCN [12].

In conclusion, recognition of SCN as a potential contributor to LBP is essential for effective treatment. Patients with SCN can present with having isolated LBP but can also demonstrate associated leg pain, which increases the diagnostic challenge for physicians. A physical examination can help elucidate SCN versus other pain generators. Early detection of SCN can help prevent unnecessary lumbar procedures or surgeries. PNS can be an effective long-term solution for patients with chronic SCN who have been unresponsive to previous treatment modalities.

## Conclusions

Superior cluneal neuralgia should be considered in the differential for patients with LBP with or without leg involvement. The risk factors for superior cluneal neuralgia include conditions or procedures that can increase myofascial compression, such as fibrosis following spinal or gluteal procedures or maladaptive posturing due to pain or other medical conditions. Peripheral nerve stimulation can be an effective long-term treatment strategy for the management of superior cluneal neuralgia.
